# A Machine Learning Framework for Gait Classification Using Inertial Sensors: Application to Elderly, Post-Stroke and Huntington’s Disease Patients

**DOI:** 10.3390/s16010134

**Published:** 2016-01-21

**Authors:** Andrea Mannini, Diana Trojaniello, Andrea Cereatti, Angelo M. Sabatini

**Affiliations:** 1 The BioRobotics Institute, Scuola Superiore Sant’Anna, Pisa 56127, Italy; sabatini@sssup.it; 2 Information Engineering Unit, POLCOMING Department, University of Sassari, Sassari 07100, Italy; dtrojaniello@uniss.it (D.T.); acereatti@uniss.it (A.C.)

**Keywords:** gait classification, wearable sensors, inertial sensors, hidden Markov model, elderly, hemiparetic, Huntington’s disease

## Abstract

Machine learning methods have been widely used for gait assessment through the estimation of spatio-temporal parameters. As a further step, the objective of this work is to propose and validate a general probabilistic modeling approach for the classification of different pathological gaits. Specifically, the presented methodology was tested on gait data recorded on two pathological populations (Huntington’s disease and post-stroke subjects) and healthy elderly controls using data from inertial measurement units placed at shank and waist. By extracting features from group-specific Hidden Markov Models (HMMs) and signal information in time and frequency domain, a Support Vector Machines classifier (SVM) was designed and validated. The 90.5% of subjects was assigned to the right group after leave-one-subject–out cross validation and majority voting. The long-term goal we point to is the gait assessment in everyday life to early detect gait alterations.

## 1. Introduction

Wearable inertial sensors can be used to analyze human physical activity for prolonged periods of time and with minimal subject’s discomfort. Within this context, the assessment of gait in terms of quality and quantity is of great relevance since it provides indications of the level of physical mobility, of the risk of fall or of the effects of a therapy [1]. The development of automatic methods to discriminate between normal and abnormal gait and among different pathological gait patterns would be of great interest in several clinical applications, [2,3]. This, along with the possibility to collect a large amount of data during everyday life conditions, may open up new perspectives for the early identification of gait disorders and for the implementation of general pre-screening procedures.

In general, pathological gaits can be classified based on their characteristic motor features and according to the dominant observed motor disturbance [4]. The fundamental prerequisite for the implementation of automatic methods for the classification of gait disorders is the possibility to identify selected gait features for which the *intra-pathology* variability is smaller than the *inter-pathology* variability.

Inertial measurement units (IMUs) including combinations of accelerometers and gyroscopes have been successfully used for assessing gait characteristics (*i.e.*, gait spatio-temporal parameters, gait variability) and the quality and quantity of physical activity in both healthy and pathological populations [5,6,7]. Recently, novel IMU-based approaches based on the statistical modeling of gait sequences using Hidden Markov Models (HMMs) have been proposed [8,9,10,11,12,13,14,15,16]. The inclusion of the statistical information on the signal morphology in addition to the signal value itself (the latter is of main interest in, e.g., classical threshold-based methods) was exploited to model gait data for both segmentation and classification purposes [8,10]. Some examples of recent applications of this statistically-intensive approach include discrimination between walking and jogging [8], classification of type of walking (level walking, inclined walking and stair climbing) [17], gesture recognition [18] and user authentication based on gait [19]. Machine learning methods such as artificial neural networks and support vector machines (SVM) found application in automatic recognition of pathological gait. Lakany proposed a solution for pathology detection from kinematic data obtained using stereo-photogrammetry during gait [20]. In particular, an artificial neural network was designed and validated to recognize 89 control subjects from 32 pathological patients affected by different gait disorders (cerebral palsy, polio or spina-bifida), using features extracted from spatial and temporal gait parameters. Begg and colleagues applied SVM classifiers to recognize gait changes due to ageing from kinematic data. A dataset of gait measurements of 30 young and 28 elderly subjects obtained from stereo-photogrammetry was considered, extracting features from foot clearance [21]. In another study, Pogorelc and colleagues compared several classifiers including neural networks and SVM to automatically recognize five healthy controls and four patients with hemiplegia using kinematic data [22]; features were extracted from joint angles, relative displacements, left-right symmetry and walking speed.

A few studies have attempted to categorize abnormal gait patterns from IMU data. Although no classification tests were conducted, Abaid and colleagues found significant differences in the duration of the gait phases between 10 healthy and 10 hemiplegic children using IMU data [14]. Chen and colleagues proposed an HMM based approach for discriminating between two simulated gait deviations (toe-in and toe-out gait) [12]. However, the method was applied and validated on four healthy subjects only, who were asked to reproduce the two abnormal gait conditions.

The objective of the current work is to propose and validate a machine learning framework for the definition of features to be used for the classification of different pathological gaits from IMU data. Specifically, this work presents a methodology based on the training of class-specific HMMs used to recognize the pathology group jointly with SVM classifiers. This mixed approach should take advantage of each single methodology. In fact, previous HMM based solutions have shown the capability to capture gait characteristics useful for classification [8,14], whereas gait classification studies involving SVM classification have reported high rates in recognizing gait alteration [21,22,23].

Very often the validation phase of newly proposed methodologies for the gait classification, especially in pathological populations, is overlooked or based on N-fold cross-validation approaches that includes data from the same subject in both training and test sets [21,22]. In the present study, we tested the proposed methodology and validated it on about 3500 gait cycles recorded on three different populations (Huntington’s disease and post-stroke subjects and healthy elderly controls) [15]. The methodology validation was performed using a Leave-One-Subject-Out (LOSO) cross-validation approach, so as to properly assess the algorithm behavior when evaluated on a new subject. The two pathological populations were selected as a paradigm since they are characterized by gait patterns that result in IMU signals with characteristic features [24,25,26,27,28].

The main motivation of this work is to lay the methodological foundation for mobile-based gait assessment tools, to be possibly included in health-tracking platforms such as *Google Fit* or *Apple Health*. The long-term goal we point to is then *the gait assessment in everyday life*, in the attempt to detect gait alterations early.

## 2. Materials and Methods

### 2.1. Subjects

The study included 15 post stroke patients (PS) (five females, ten males; mean (sd) age: 61.3 (13) y.o., height: 1.71 (0.06) m, mass: 78.7 (16.3) kg), 17 subjects with Huntington’s disease (HD) (seven females, ten males; mean (sd) age: 54.3 (12.2) y.o., height: 1.66 (0.08) m, mass: 61.5 (10.3) kg), and 10 healthy elderly (EL) (six females, four males; mean (sd) age: 69.7 (5.8) y.o., height: 1.62 (0.08) m, mass: 63.6 (5.7) kg). Subjects were enrolled from the out-patient Movement Disorders Clinic of the University of Genoa. Disease severity was determined by means of the Functional Ambulatory Category (FAC) which rates gait from 5 (independent walker) to 1 (walks with physical assistance only) [29] for the PS subjects (3.2 ± 1.5) and the Unified Huntington’s Disease Rating Scale (UHDRS) [30] for the HD subjects (40 ± 20). The UHDRS scale quantifies the general level of impairment taking into account motor, cognitive and behavioral aspects. Because the focus of the present study is on gait only, information related to ambulation only was considered (normal walking and tandem walking) to define a more specific scale named HDRS’. The level of the locomotor disability decreases with the scale score (8: not ambulating–0: normal gait). The Declaration of Helsinki was respected, all subjects provided informed written consent, and local ethic committee approval was obtained.

### 2.2. Acquisition Protocol

Three IMUs (Opal, APDM, Inc., Portland, OR, USA) featuring a tri-axial accelerometer and a tri-axial gyroscope (unit mass 22 g, unit size 48.5 mm × 36.5 mm × 13.5 mm, sampling frequency 128 Hz, accelerometer range ± 6 *g*, where *g* = 9.81 m/s^2^) were located on both shanks (about 20 mm above the malleoli with *x*, *y* and *z* axes pointing in vertical (VT, downward), antero-posterior (AP, forward) and medio-lateral (ML, right), directions respectively) and over the subject’s lumbar spine, between L4 and S2, of each participant. Although this work did not include anatomical calibration of the IMU positioning [31,32,33,34], care was taken to align the IMU axes to the anatomical axes. IMUs signals were low-pass filtered using a double-pass second order Butterworth filter with 5Hz cut-off frequency. A seven-meters long instrumented gait pressure mat (GAITRite™ Electronic Walkway, CIR System Inc., Franklin, NJ, USA) acquired at 120 Hz (spatial resolution accuracy: ±12.7 mm; time accuracy: ±1 sample) was used to acquire reference data. The instrumented mat returned the foot strike (FS) and toe off (TO) events and all relevant gait temporal parameters. The IMUs and the instrumented mat were synchronized (± 1 sample) by means of a wired connection between the IMU access point and the sensorized mat. The synchronization error was then evaluated in preliminary tests using the signals generated when a wooden cane, instrumented with one IMU, impacted the sensorized mat.

Subjects were asked to walk back and forth for about one minute along a 12-m walkway with the instrumented mat placed two meters from the starting line. Subjects walked both at self-selected comfortable speed and higher speed, wearing their own shoes. Each passage on the instrumented mat was considered as a separate gait trial.

### 2.3. Gait Classification Strategy

**Figure 1:**
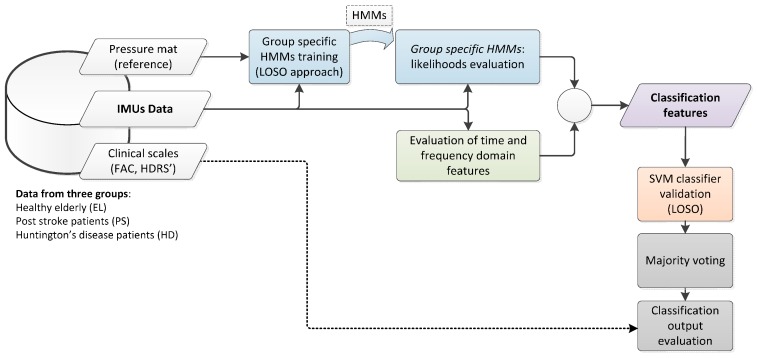
General block scheme of the algorithm for gait classification.

A classification strategy was implemented for the automatic identification of pathological gaits based on IMU data, as reported in Figure 1. The classification framework was defined through three major steps: (i) the classification process started from implementing class-specific HMMs, whose likelihoods of observing data given each model were evaluated; (ii) the HMM likelihoods were included in a wider feature set, which was then classified using an SVM classifier; (iii) a majority voting (MV) classification post-processing was cascaded to summarize the results obtained in step *ii*.

To evaluate the performance of the proposed classification approach, a LOSO cross-validation was carried out. It consists of training models using all data except those from the subject being tested and then repeating the evaluation for all the subjects in the dataset. Such a validation approach is particularly useful in evaluating the inter-subject generalization capability of the proposed methodology [8,35]. The criterion for assessing the quality of classification was given by the correct classification accuracy resulting from LOSO cross-validation. The accuracy was evaluated from the obtained confusion matrices.

The methodology was validated offline, by running all tests on a personal computer running Matlab (vers 2013b, the MathWorks, Natick, MA, USA). However, the online implementation of the classification method looks feasible, given that it would only require feature extraction and SVM testing, which present, respectively, time complexity of O(NlogN) and O(N^2^). SVM classification and HMM-based gait assessment were successfully tested on Android-based smartphones in some previous works of ours [36,37].

#### 2.3.1. Classification Using Group-Specific HMM Likelihood

HMM is a double stochastic process in which the existence of a set of discrete states is assumed for a given system. The first stochastic process describes how the system may jump from one state to another (transition probability), under the hypothesis that the next state depends only on the state at the present time (Markov property). The state sequence is not observed—it is hidden to the observer, who has access to the emissions of each state only (in practical terms, the emissions can be considered as the observed quantities such as sensor data that are used to infer the hidden structure of the modeled phenomenon). The second stochastic process yields the statistical description governing the emissions of each observed variable (emission probability), in terms of either discrete probabilities or probability density functions. HMMs have been applied to solve classification problems thanks to their capability of summarizing signals’ characteristics using few parameters [38]. For each class, a specific model was trained. New data were then classified by evaluating which of the available models better explains data itself (likelihood) [38]. Given the cyclical nature of gait, we adopted a left–right HMM characterized by two states paired to the stance and swing phases as defined by the TO and FS events. The two state models were trained in a supervised way using the FSs and TOs annotations provided by the instrumented mat, used as a *gold standard*.

The proposed set of HMM emissions was composed of seven observed variables. Six elements were computed from a single shank sensor according to a previous work on gait segmentation [15]: ML angular velocity and its approximated derivative, AP acceleration and its approximated derivative, and approximated derivative of the ML and VT accelerations. The seventh element is selected from waist data (ML acceleration). The seven elements of data sequences will be referred to as *channels* in the text. Emission probabilities were modeled in the HMMs as Gaussian mixtures with three modes. To use all the experimental data available, we considered the right and left shank sensors as two different sources. Therefore, for each passage on the instrumented mat, two sets of experimental observations were available. The sign of the waist ML acceleration was changed when organizing emission vectors for the left side shank. This was done to have the same sign in the representation of waist data for both sides. The impaired side in PS subjects was also annotated.

**Figure 2:**
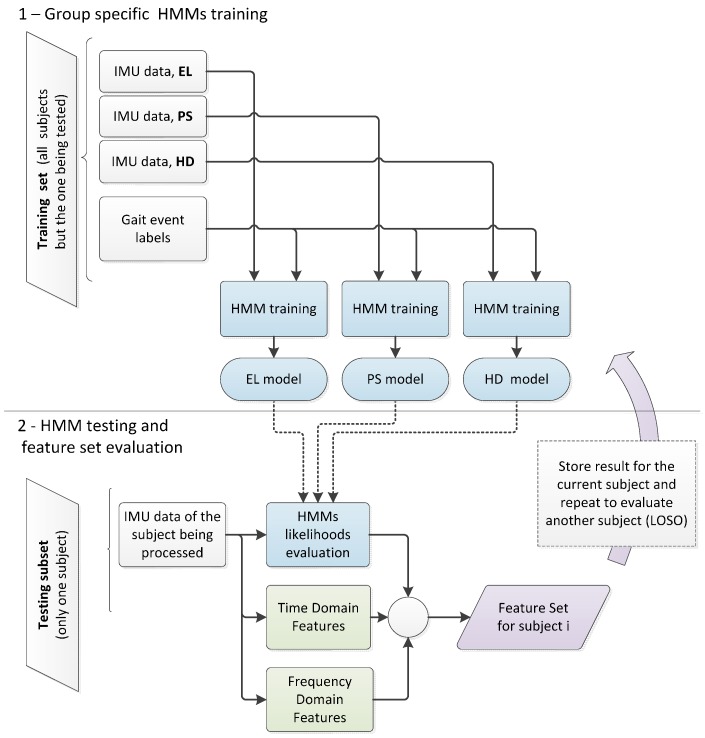
Feature set definition for classification. The information from the subject being tested was not included in the training set.

For each group analyzed (EL, PS, HD), a specific model was trained using the experimental data recorded for the relevant group. The training was supervised using the approach reported in [39]. Class-specific models were then used to classify new experimental data, as shown in Figure 2. The forward-backward algorithm, implemented as it was described in the work byRabiner and colleagues [38], was applied to evaluate the likelihoods of observing data given each class-specific model. HMM log-likelihoods at each validation step were evaluated on a data window of the same length, corresponding to the first 2 s from each passage on the sensing mat. The natural logarithm was applied to likelihoods, so as to obtain log-likelihoods [38]. The classification output for each passage was obtained by selecting the model showing the highest log-likelihoods.

#### 2.3.2. SVM Classifier

SVM is a geometric-based classifier that constructs boundaries maximizing the margins between the nearest features relative to two distinct classes [40]. An excellent description of SVM algorithms can be found in the works by Vapnik and colleagues [40]. In this work, in particular, we used the LibSVM implementation of these algorithms [41].

To use a more exhaustive feature set, we added to the group-specific HMM log-likelihoods three features obtained by subtracting pairs of group specific likelihoods. In this case, in order to account for different information as above, likelihoods were evaluated on data from the *whole* duration of the passage on the instrumented mat. These three features were added to extract additional information from HMM models. In particular, the differences between log-likelihoods were proposed to obtain features that hold information about binary classification between two of the available groups. Since the value of the likelihood is known to depend on the length of the sequence, the difference between log-likelihoods for two models helped analyze data from different trials of different length. Moreover, twelve additional time and frequency domain features extracted from the IMU data were included (Table 1); such features confirmed their capability to extract information about ambulation in previous studies on data classification using inertial sensors [23,42].

**Table 1:** Features for classification.

**HMM-Based Features**	H1. Log-likelihood, EL model (limited to a 2-s window)	
	H2. Log-likelihood, PS model	
	H3. Log-likelihood, HD model	
	H4. Difference between log-likelihoods given EL and PS models (for all available data)	
	H5. Difference between log-likelihoods given EL and HD models	
	H6. Difference between log-likelihoods given PS and HD models	
**Time Domain Features**	T1. Mean value	Evaluated for each channel (84 features)
	T2. Standard deviation	
	T3. Variance	
	T4. Maximum	
	T5. Minimum	
	T6. Range	
**Frequency Domain Features**	F1. Power at first dominant frequency (P1)	
	F2. Power at second dominant frequency	
	F3. First dominant frequency	
	F4. Second dominant frequency	
	F5. Total power (PT)	
	F6. P1/PT	

The complete features data set hence included 90-dimensional feature vectors (six time domain features for each channel, six frequency domain features for each channel and six HMM-based features). An SVM classifier was then cascaded to classify feature vectors within the three considered classes (EL, PS and HD). Radial basis function kernels were used and SVM kernel parameters were optimized by running a grid search across parameter configurations (upper complexity bound *C* and kernel variability *γ*). For each of the tested feature subsets, the optimization criterion was the maximization of the minimum class-specific accuracy.

**Figure 3:**
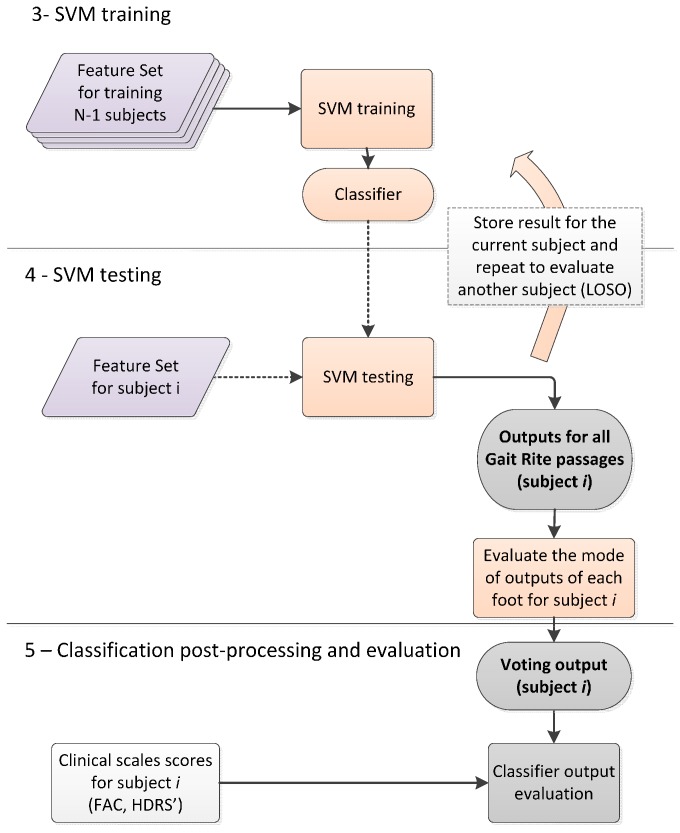
Block scheme of the SVM classifier validation. The LOSO approach was followed: data from *N*-1 subjects were used for training and the obtained classifier was tested on the features from the remaining subject.

It is important to notice that both group-specific HMMs and SVM classifiers were evaluated using an LOSO cross-validation approach. This implies that at each validation step, namely for each subject to be evaluated, three HMMs were trained to evaluate likelihoods and a SVM classifier was trained using the feature set described in Table 1, as it is summarized in Figure 2 and Figure 3.

#### 2.3.3. Classification Post-Processing with Majority Voting

To improve the final prediction and summarize the results, a majority voting (MV) strategy was finally applied [43]. MV is a post-processing technique applied to the classification outcomes, where multiple classification outcomes are merged to generate a single classification outcome. In MV, each classification outcome generates a ‘‘vote’’ for the corresponding class; the class collecting more votes is then selected as the winner of the poll.

In this paper, a vote was assigned for each subject, passage on the instrumented mat and side. For each subject, votes for each passage and side were analyzed to generate the assignment of the subject to the corresponding class. A slightly different approach was applied to PS patients, since, for them, a distinction between impaired and not-impaired side is relevant. For this group, instead of summing up votes of both sides to get a unique classification outcome, as it was done for EL and HD groups, the MV was applied, separately to the votes collected for each side.

### 2.4. Data Analysis

For each subject group (EL, PS, and HD), the accuracy of the classification obtained by solving the classification problem using (i) the HMM-based information only; and (ii) the SVM classifier output were computed. For the latter approach, the influence of the features on the classification output was also investigated. In particular, three different sets of features were compared: (a) HMM-based features; (b) time and frequency domain features; (c) full features set. The results provided by (i) and (ii) were compared with those obtained after (iii) MV was applied using the three sets of features previously described, Table 1.

The relationships between classification outputs and clinical scales were investigated by correlating the majority votes with the clinical scales values. The FAC levels were used for PS patients, whereas HDRS’ scores were used for HD patients.

## 3. Results

The number of passages on the instrumented mat varied from subject to subject from a minimum of two passages to a maximum of 16, in relation to the average gait speed, Table 2. The complete dataset analyzed consisted of 390 passages on the sensorized mat; for each passage, 4.5 strides were counted, on average, for each foot.

**Table 2:** Dataset details.

Group	Number of Passages					Number of Strides Per Passage				
	Min	Max	Mean	SD	Total	Min	Max	Mean	SD	Total
EL	6	15	12.4	3.1	124	2	5	3.5	0.7	862
PS	2	11	6.3	3.1	95	3	24	6.0	3.6	1144
HD	4	16	10.1	3.4	171	2	21	4.3	2.6	1467
Overall	2	16	9.3	4.0	390	2	24	4.5	2.7	3473

### Gait Classification

Results for the automatic classification obtained by group-specific HMMs are reported in Part 1 of Table 3. The overall accuracy was 66.7%. Most data were incorrectly classified as HD data.

After applying SVM classification to the features set of Table 1, the results improved significantly. As reported in Parts 2A, 2B and 2C of Table 3, the bias toward the HD class was reduced by applying SVM classification. In particular, by limiting the SVM classifier to HMM-based features, an overall accuracy of 71.5% was obtained (Part 2A of Table 3). Similarly, by using time and frequency domain features only, the overall recognition accuracy was 71.7% (Part 2B of Table 3). The inclusion of the full feature set resulted in a classification accuracy of 73.3%, as it is shown in Part 2C of Table 3. After the MV was applied, by using all the features, the 90.5% of the subjects were correctly classified (Table 4). For PS patients, in order to discriminate between impaired and not impaired side, two results for each subject are reported in Table 3 and Table 4. As it is shown in Table 4, Part 2C, there were no misclassifications from HD or PS classes to the EL class, and then misclassifications occurred only between the two pathological conditions (PS and HD).

**Table 3:** Confusion matrices. Results are reported for two different strategies: (1) solving the classification problem using Hidden Markov Models (HMM) based information only; (2) solving the problem using a Support Vector Machine (SVM) classifier. In the latter case, the contributions of HMM-based features only (2A), of time and frequency domain features only (2B) and of the full features set (2C) are presented separately. Confusion matrices refer to the classification of single mat passages: each entry in the matrix corresponds to a passage on the mat of one foot. The amount of passages is then doubled with respect to data in Table 2. Correct classifications are in bold.

		Classification Output		
		EL	PS	HD
*1. HMM-based information (maximum log-likelihood)*				
**Actual Label**	**EL**	**176 (71%)**	0 (0%)	72 (29%)
	**PS–not imp. side**	0 (0%)	**16 (16.8%)**	79 (83.2%)
	**PS–imp. side**	0 (0%)	**51 (53.7%)**	44 (46.3%)
	**HD**	60 (17.5%)	5 (1.5%)	**277 (81%)**
		Overall accuracy 66.7% of mat passages		
*2A. SVM classifier (HMM-based features only)*				
**Actual Label**	**EL**	**168 (67.7%)**	50 (20.2%)	30 (12.1%)
	**PS–not imp. side**	11 (11.6%)	**69 (72.6%)**	15 (15.8%)
	**PS–imp. side**	0 (0%)	**93 (97.9%)**	2 (2.1%)
	**HD**	55 (16.1%)	59 (17.3%)	**228 (66.7%)**
		Overall accuracy 71.5% of mat passages		
*2B. SVM classifier (time and frequency domain features only)*				
**Actual Label**	**EL**	**182 (73.4%)**	0 (0%)	66 (26.6%)
	**PS–not imp. side**	3 (3.2%)	**57 (60%)**	35 (36.8%)
	**PS–imp. side**	1 (1.1%)	**78 (82.1%)**	16 (16.8%)
	**HD**	63 (18.4%)	37 (10.8%)	**242 (70.8%)**
		Overall accuracy 71.7% of mat passages		
*2C. SVM classifier (all available features)*				
**Actual label**	**EL**	**159 (64.1%)**	48 (19.4%)	41 (16.5%)
	**PS–not imp. side**	0 (0%)	**71 (74.7%)**	24 (25.3%)
	**PS–imp. side**	0 (0%)	**88 (92.6%)**	7 (7.4%)
	**HD**	39 (11.4%)	49 (14.3%)	**254 (74.3%)**
		Overall accuracy 73.3% of mat passages		

**Figure 4:**
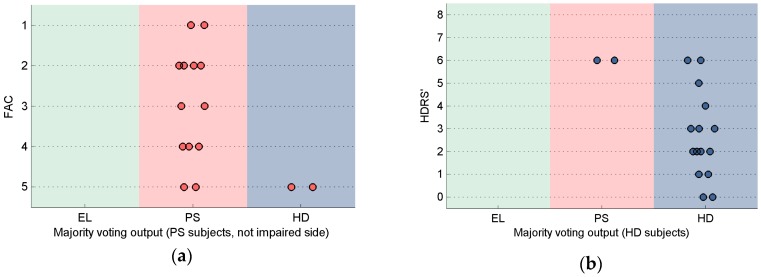
Output of the classifier after MV in relation to clinical scales: (**a**) PS subjects (impaired side only), in relation to the FAC scale. No data were misclassified from the PS class to the EL class. Two subjects are misclassified from the PS class to the HD class; (**b**) HD subjects in relation to the HDRS’ scale. No data were misclassified from the HD class to the EL class. Two subjects were misclassified from the HD class to the PS class.

The evaluation of classifiers with respect to the clinical scales values is summarized in Figure 4.

Figure 4a concerns PS subjects and reports only results for the not impaired side given that the classification showed 100% accuracy for the impaired side. In general, FAC values for data correctly classified as PS were low, whereas misclassifications from PS to the EL class showed higher FAC values, as shown in Figure 4.

A relation was also found between classifier output and the HDRS’ values, showing higher values, on average, for data misclassified as PS, Figure 4b.

**Table 4:** Confusion matrices obtained after majority voting (MV). Results are reported for two different strategies: (1) solving the classification problem using HMM based information only; (2) solving the problem using a SVM classifier. In the latter case, the contributions of HMM-based features only (2A), of time and frequency domain features only (2B) and of the full features set (2C) are evaluated separately. Each entry in the matrix corresponds to a subject. Post stroke (PS) subjects were reported twice, separating the two sides contributions. Correct classifications are in bold.

		Classification Output		
		EL	PS	HD
*1. HMM-based information (maximum log-likelihood)*				
**Actual Label**	**EL**	**7 (70%)**	0 (0%)	3 (30%)
	**PS–not imp. side**	0 (0%)	**5 (33.3%)**	10 (66.7%)
	**PS–imp. side**	0 (0%)	**10 (66.7%)**	5 (33.3%)
	**HD**	1 (5.9%)	0 (0%)	**16 (94.1%)**
		Overall accuracy 76.2% of subjects		
*2A. SVM classifier (HMM-based features only)*				
**Actual Label**	**EL**	**9 (90%)**	0 (0%)	1 (10%)
	**PS–not imp. side**	1 (6.7%)	**13 (86.7%)**	1 (6.7%)
	**PS–imp. side**	0 (0%)	**15 (100%)**	0 (0%)
	**HD**	1 (5.9%)	4 (23.5%)	**12 (70.6%)**
		Overall accuracy 85.7% of subjects		
*2B. SVM classifier (time and frequency domain features only)*				
**Actual Label**	**EL**	**8 (80%)**	0 (0%)	2 (20%)
	**PS–not imp. side**	0 (0%)	**13 (86.7%)**	2 (13.3%)
	**PS–imp. side**	0 (0%)	**14 (93.3%)**	1 (6.7%)
	**HD**	3 (17.6%)	0 (0%)	**14 (82.4%)**
		Overall accuracy 83.3% of subjects		
*2C. SVM classifier (all available features)*				
**Actual label**	**EL**	**9 (90%)**	1 (10%)	0 (0%)
	**PS–not imp. side**	0 (0%)	**13 (86.7%)**	2 (13.3%)
	**PS–imp. side**	0 (0%)	**15 (100%)**	0 (0%)
	**HD**	0 (0%)	2 (11.8%)	**15 (88.2%)**
		Overall accuracy 90.5% of subjects		

## 4. Discussion

We proposed and validated a general probabilistic modeling approach based on IMU recordings for the classification of normal and pathological gaits (EL, HD and PS). The classification framework was defined through three major steps starting from a classification based on class-specific HMMs, then including the HMM likelihoods in a wider feature set to be classified using a SVM classifier, and finally cascading MV to summarize the results obtained.

The classification based on the maximum class-specific likelihood evaluation [38], was only partially successful to solve the classification problem (overall accuracy 66.7%). The significant bias toward the HD class could be related to the large inter- and intra- subject gait variabilities that characterize the PS group [44]. In fact, if the population analyzed is characterized by a highly variable and asymmetric gait, the internal consistency of the stance and swing phases duration is likely to be poor. This circumstance can reduce the efficacy of the specific models to capture a common signature that can describe the group as a whole.

The use of SVM classifiers improved the class separation thanks to their capability to define more complex decision boundaries. Results indicated that HMM-based and time/frequency domain features perform similarly when used separately, but they improve the final outcome when merged (up to 73.3%). An explanation could be that time and frequency domain features provide information about the gait variability that are somehow complementary to those contained in the HMM likelihoods.

Finally, the MV step summarized results by smoothing the effects of intra-subject variability, providing a high overall accuracy in terms of subjects classification up to 90.5%. In fact, the large variability observed during each trial is smoothed by taking into account a larger amount of trials. It is interesting to note that, despite of a similar accuracy, before applying the MV procedure, the outputs for HMM-based features are more distributed across classes than those obtained using time and frequency domain features only. As a result, the classification based on HMM features takes more advantage from the voting procedure. It is noted that the proposed MV strategy treats each vote equally. To improve the methodology we could modify the majority voting to a weighted majority voting approach in which each vote could be weighted based on the classification uncertainty, as estimated by, e.g., cascading a logistic regression to the SVM classifier [37,45]. However, as a first step in the development of the method, the voting approach in which each vote contributes equally was preferred and any refinement of the voting procedure is then left to future developments.

Looking at the few misclassifications between PS and HD in relation to the scores of the clinical scales (Figure 4), it is noted that two PS patients with a low level of impairment (FAC = 5) were assigned to the HD group when testing their not-impaired side, whereas two HD patients with severe gait impairments (HDRS’ = 6) were classified as PS. This is somehow an expected result; in fact, the patients with clinical scores at the extreme range of the specific reference population are more likely to be misclassified. In this regard, it is important to highlight that, because the proposed classification method focus on the gait alterations caused by the pathology and not on the pathology itself, pathology misclassifications are not to be considered necessarily as errors: altered gait patterns can be common to different pathologies and mild impairments may not affect significantly gait patterns. Hence, the direct application of the proposed methodology to different pathologies that affect gait would not be straightforward. The inclusion of additional pathological populations would require the definition of disorder-specific models.

## 5. Conclusions

The present methodology allowed to properly discriminate abnormal gait patterns using an SVM classifier, taking advantage of HMM derived information. The use of complementary features (HMM likelihoods and time-frequency domain features) along with an MV classification post-processing allowed for the improvement of the classification outcomes (overall accuracy 90.5%). The few incorrect classifications were found for those patients with clinical scores at the extreme range of the specific reference population. As a paradigm for testing the performance of the classification methodology, we selected two pathological populations—Huntington’s disease and post-stroke subjects and elderly as control group. However, it is important to highlight that the same general approach can be extended to the classification of other pathological populations after those specific validations are carried out. Future works will focus on this, by extending the vocabulary of recognized conditions, improving model complexity, including gait spatial and temporal parameters as classification features and by defining metrics for gait assessment based on the same wearable sensing sources and methodological approach.
